# Attachment style moderates the relationship between social media use and user mental health and wellbeing

**DOI:** 10.1016/j.heliyon.2020.e04056

**Published:** 2020-06-05

**Authors:** Lindsay Young, Daniel C. Kolubinski, Daniel Frings

**Affiliations:** Centre for Addictive Behaviours Research, School of Applied Sciences, London South Bank University, United Kingdom

**Keywords:** Psychology, Attachment, Social networks, Depression, Anxiety, Social media

## Abstract

**Background:**

Past research has correlated social media use with a variety of mental health outcomes – both positive and negative. The current study aims to explore two possible moderators of the link between social media use and mental health outcomes; specifically, the effects of having an anxious and/or avoidant attachment style.

**Method:**

A cross-sectional correlational design was implemented. Participants (*n* = 124). aged ≥18 years completed scales measuring experiences in close relationships, general problematic Internet use, psychological wellbeing and satisfaction with life.

**Results:**

Negative relationships between problematic social media use and both psychological wellbeing and life satisfaction were observed. For psychological wellbeing, the relationship was strongest amongst individuals who were low in avoidant attachment and high in anxious attachment.

**Discussion:**

These results suggest that attachment style impacts the extent that social media affects user mental health and wellbeing; partly explaining paradoxical results in previous research.

**Conclusion:**

We suggest that individuals who are high in anxious attachment and low in attachment avoidance may be more susceptible to negative outcomes arising from problematic SNS use.

## 1 Attachment style moderates the relationship between social media use and user mental health and wellbeing

Recent years have witnessed a significant increase in social media use (Aboujaoude and Starcevic, 2015). Alongside the intended effects that social networking sites (SNSs) have in terms of developing new and maintaining existing relationships (Chen, 2019), SNS use has been linked with a variety of mental health outcomes – both positive and negative (Barry et al., 2017). The current study aims to explore two possible moderators of the link between social network use and mental health outcomes; specifically the effects of having an anxious and/or avoidant *attachment style* (the behavioural system and tendencies developed during infancy that aims to achieve psychological and physiological security (Gillath et al., 2019)).

### 1.1 Social media use, relationships and mental health

One approach to research into social media use and mental health assumed that a major user motive was to relieve psychosocial problems (Kim et al., 2009). A major implication of this research was that a focus on maladaptive use of SNS in response to stressors presented bias toward finding problematic psychological outcomes such as: addiction (Kim et al., 2009); solitary behaviour (Kraut et al., 1998); and associated mood disorders (Weinstein et al., 2015).

More recently, research has been conducted to account for this bias. For instance more recent studies have attributed no predicted effect on mental health and wellbeing as a result of social media use (Berryman et al., 2018). Likewise, research has also explored how social media use can also be attributed to positive user mental health. In fact, literature has since highlighted a direct contrast to earlier research (Kraut et al., 1998), demonstrating how relationships and communication have benefited from SNS use (Nowland et al., 2018). This may be in part because SNSs provide even the most insecurely-attached individuals with a place to safely develop social relationships and maximise social networks, resulting in more peer support and increased wellbeing (Ahn and Shin, 2013; Bessière et al., 2008; Kraut et al., 2002).

SNSs have also been found to provide immersive, involving and stimulating environments for users (Vaghefi and Lapointe, 2013). Such research has suggested that ‘loneliness’ *determines* SNS use rather than it being an outcome (Nowland et al., 2018). For instance, Shaw and Gant (2002) show that social media usage significantly decreases self-reported incidents of loneliness and depression, while users also experienced a significant increase in self-esteem. Additionally, SNSs have also been linked with decreased self-reports of depression and loneliness demonstrating SNSs as buffers for mental health difficulties and stressful offline life events (Caplan et al., 2009; Shaw and Gant, 2002).

One limitation of previous research in this area is that the majority of studies have reduced the operationalisation of mental health to depression, stress and/or loneliness (Dhir et al., 2018). Perhaps as a result, previous research has yielded mixed and inconclusive results. For instance, one meta-analysis concluded that SNS use was positively correlated with an increase in civic engagement and social capital (Skoric et al., 2016). In contrast, another concluded that time spent on social media was positively correlated with problematic outcomes such as addictive behaviour and a decline in social capital (Kim et al., 2009). A current debate between Twenge and Campbell (2019) and Orben and Przybylski (2019) highlights the current conflict in understanding the effects of social media use on user mental health and wellbeing. On one hand, Twenge and Campbell (2019) has overtly suggested correlations between increased social media use and increased depression, suicidal ideation and declining wellbeing. In contrast, Orben et al.'s (2019) findings suggest that social media use alone barely affects user mental health and wellbeing and instead such effects are nuanced by other variables such as gender and analytical methods. This reinforces the notion that past and current findings are inconsistent and limited to description and exploration, where a direct linear relationship between SNS use and the dependent variable, such as depression or loneliness, is assumed, rather than testing the boundary conditions which dictate a bi-directional and dynamic relationship (Nowland et al., 2018). The current study proposes that the intent associated with SNS use and associated outcomes are dependent on other psychosocial or individual-level factors. In particular, our focus lies with *attachment style* as a moderating variable.

### 1.2 Attachment style and SNS use

Contemporary models of adult attachment conceptualise individual differences in attachment as variations along two orthogonal and continuous dimensions; attachment anxiety (how fearful one is of rejection – characterised by the level of demanding or ‘needy’ behaviour) and attachment avoidance (how comfortable or uncomfortable one is with closeness, exhibited through passive and withdrawn behaviour; Brennan et al., 1998; Fraley et al., 2015). This dimensional perspective of attachment has been less studied alongside online, in contrast to offline, social relationships. This is despite differences between the two modes, particularly around reduced geographical boundaries, reduced need for favorable aesthetics, greater control over dedicated time to interactions and increased anonymity.

McKenna and Bargh (1999) argue that these factors mean that individuals will use SNS for different reasons. It was inferred that social media use was a way for individuals with high attachment anxiety to manage self-presentation and obtain desired intimacy while controlling for risks of rejection (Coan and Sbarra, 2015; Oldmeadow et al., 2013). Interestingly, individuals with high attachment avoidance have previously reported to be either affected negatively by social networking or not at all (Hart et al., 2015; Oldmeadow et al., 2013). Evidence suggests that anxiously attached individuals are more likely to use social networking sites than those who are highly avoidant (Chen, 2019) possibly because social media allows anxiously attached individuals to appear popular, leading to a positive impact on user wellbeing (Oldmeadow et al., 2013).

### 1.3 Attachment Style, Social Media and Mental Health

Differences in attachment style have been shown to influence SNS use, emotional regulation and psychological functioning (Lin, 2015; Marganska et al., 2013). Previous research suggested that anxiously attached individuals use social media to avoid more personal offline forms of communication; implying that anxiously attached individuals utilise social networking sites to maintain relationships at a psychological arm's length (Nitzburg and Farber, 2013). One outcome of using social media for anxiously attached individuals is reported feelings of perceived communion, interpreted to reflect needs for intimacy from others (Shaver and Mikulincer, 2002). Likewise, such exposure to perceived communication has been associated with higher ratings of self-esteem (Carnelly and Rowe, 2007). Hence, anxious attachment has been previously linked with self-reported improvements in mental health through social media use (Lin, 2015).

Further contradictory findings have been produced when studying insecurely attached individuals. For instance, the passive consumption of other people's lives via social media was significantly correlated with a decreased satisfaction of live for insecurely attached individuals in general (Krasnova et al., 2013). Specifically, Wei et al. (2005) suggest that highly avoidant individuals, in particular, experience loneliness through self-employed segregation and fear of rejection in both offline and online capacities. Likewise, higher attachment anxiety scores are associated with negative self-conceptions (McWilliams and Asmundson, 2007) falling in line with the deindividualisation theory that predicts a psychological state of decreased self-evaluation when social comparison is reported (Stronge et al., 2015).

High attachment avoidance has been associated with negative representations of others and deactivation of the attachment system altogether whereby individuals no longer seek out social relationships (Yaakobi and Goldenberg, 2014). Despite this, avoidantly-attached individuals have also reported increased levels of wellbeing as a result of SNS, because they are able to maintain relationships while still avoiding intimacy and closeness (Blackwell et al., 2017). Similarly, anxiously attached individuals have reported experiencing a perceived sense of belonging and popularity (Mikulincer et al., 2017) as a result of SNS use. Given this, one possibility is that negative mental health outcomes are greatest for people who have both high attachment anxiety and low avoidance, because such individuals are more receptive to hyper-activating attachment strategies where they seek social relation and are less likely to elude closeness (Worsley et al., 2018). From this perspective, attachment style can be conceptualised as a *moderator* of the relationship between SNS and outcomes, rather than sole a predictor of use.

### 1.4 Current study

Given findings that suggest that attachment style is a key predictor of engagement with SNS, and that both attachment style and SNS are linked to various mental health outcomes, it is also possible that the two interact. With some literature suggesting a bidirectional relationship between social media use and user mental health and wellbeing, the current study aimed to explore if the strength of the possible relationship between SNS and mental health outcomes are moderated by having (or not) anxious and/or avoidant attachment styles. Specifically, it was hypothesised that user mental health and wellbeing would be positively related to problematic SNS use when users report high attachment anxiety and low attachment avoidance.

## 2 Method

### 2.1 Participants

124 participants were recruited by advertising participation for the study on social networking sites such as; Facebook, Twitter, Instagram, LinkedIn and Snapchat. The sample was mainly Non-Hispanic White or Euro-American females (81%) with 19% of the sample being of other ethnic backgrounds. The mean age of the final sample was 30.58 years old (SD = 12.01) with 99 females and 25 males.

### 2.2 Design

A cross sectional design was employed. All participants completed online survey items published on an online data collection platform (Qualtrics). Variables measured were attachment style, SNS use, mental health and general wellbeing.

### 2.3 Materials and procedure

#### 2.3.1 Demographics

The survey included questions related to participant's socio-demographic characteristics such as their age, gender and nationality.

#### 2.3.2 Measurements

##### 2.3.2.1 Psychological wellbeing

General wellbeing was assessed using the Patient Health Questionnaire (PHQ-9; Kroenke and Spitzer, 2002). All 9 items were rated using a 4-point Likert type scale (0 = Not at all, 1 = Several days, 2 = More than half the days, 3 = Nearly every day). The presence of a major depressive episode was determined using the criteria defined in the PHQ-9 scoring instructions (based on DSM-IV-TR criteria), where scores of 15 and above out of 27 were indicative of depression symptomology. One extra question was added, “How often do you experience psychotic symptoms, such as hallucinations or delusions?” to identify and control for participants whose data may lack reliability. Cronbach's α was .88.

##### 2.3.2.2 Satisfaction with life

The Satisfaction with Life Scale (SwLS), as developed by Diener et al. (1985), was used to assess the participant's satisfaction with their own life to portray their mental health at the time of completion. It consisted of 5 items rated on a 7-point Likert scale (0 = Strongly disagree, 1 = Disagree, 2 = Slightly disagree, 3 = Neither agree or disagree, 4 = Slightly agree, 5 = Agree, 6 = Strongly agree) with the highest possible score being 30, low scores on the SwLS indicated lower satisfaction with one's life. Cronbach's α coefficient was .88.

##### 2.3.2.3 Attachment style

Attachment anxiety and avoidance were measured using the Experiences in Close Relationships-Revised Questionnaire (ECR-R; Fraley et al., 2011). The ECR-R was comprised of 18 items for measuring *attachment anxiety* (e.g. “I'm afraid that I will lose my partner's love.”) and 18 items measuring *attachment avoidance* (e.g. “I get uncomfortable when my partner wants to be very close.”). All items were rated using a 7-point Likert-type scale (0 = Strongly disagree, 1 = Disagree, 2 = Slightly disagree, 3 = Neither agree or disagree, 4 = Slightly agree, 5 = Agree, 6 = Strongly agree). Cronbach's αs were .92 for the anxiety scale and .93 for the avoidant scale. Prior to analyses, items were reverse scored as appropriate. Higher scores indicate higher levels of anxiety or avoidance.

##### 2.3.2.4 SNS use

SNS use was measured using 3, 5 and, 6-point Likert scales as well as open questions (Hughes et al., 2012; Moore and McElroy, 2012; Oldmeadow et al., 2013). Questions included: ‘‘Which of the following social networking websites do you currently have an account with?’’ (Facebook, Twitter, Instagram, Snapchat, LinkedIn, Other); “In a day, how likely are you to use social networking websites?” (1 = Extremely likely, 2 = somewhat likely, 3 = Neither likely nor unlikely, 4 = Somewhat unlikely, 5 = Extremely likely) ‘‘In a typical day, which of the following social networking websites do you use most often?’’ (1 = Facebook, 2 = Twitter, 3 = Instagram, 4 = Snapchat, 5 = LinkedIn 6 = Other); “In a typical day, about how much time do you spend using social media?”; ‘‘How many friends do you currently have on social networking websites?’’; “About how many of your friends on social networking websites have you met in person?”; and ‘‘Do you think that you spend less time socialising offline than you would if you didn't have access to social media?’’ (1 = Yes, 2 = maybe, 3 = no). Items were referenced to individually for analysis.

##### 2.3.2.5 General problematic internet use

Problematic social media use was measured using the General Problematic Internet Use Scale (Caplan et al., 2009). The scale was adapted to focus on social media rather than general Internet use by adapting all 15 items to read ‘social media’ instead of ‘Internet’. The items were rated on a 7-point Likert type scale (0 = Definitely Disagree, 1 = Disagree, 2 = Slightly disagree, 3 = Neither agree or disagree, 4 = Slightly agree, 5 = Agree, 6 = Strongly agree). The scale included 5 dimensions: *preference for online interaction* (e.g. “I prefer to use social media rather than interact with my peers face-to-face”); *mood regulation* (e.g. “I use social media when I feel down”); *cognitive preoccupation* (e.g. “I feel restless and/or frustrated when social media is unavailable”); *compulsive use* (e.g. “I rush through work responsibilities to use social media”); and *negative outcomes* (e.g. “Social media use has created problems in my life”). Higher scores on the scale were indicative of higher levels of problematic Internet use. Cronbach's α for the GPIUS was .92.

### 2.4 Procedure

The London South Bank University Research Ethics Panel provided ethical approval and oversight for the study. Following consent, participants completed the scales in the order presented above. They were then thanked and debriefed. Data were collected using an online data collection platform (Qualtrics).

## 3 Results

75% of the sample reported that they were extremely likely to use social networking sites daily with 47% of the sample using Instagram most and 45% of the sample using Facebook most frequently. While participants reported that, on average, they only knew up to 70% of individuals that they were connected with on social media, it was also revealed that 64% of the sample reported they would spend more time socialising offline if they didn't have access to social media.

**Figure 1:**
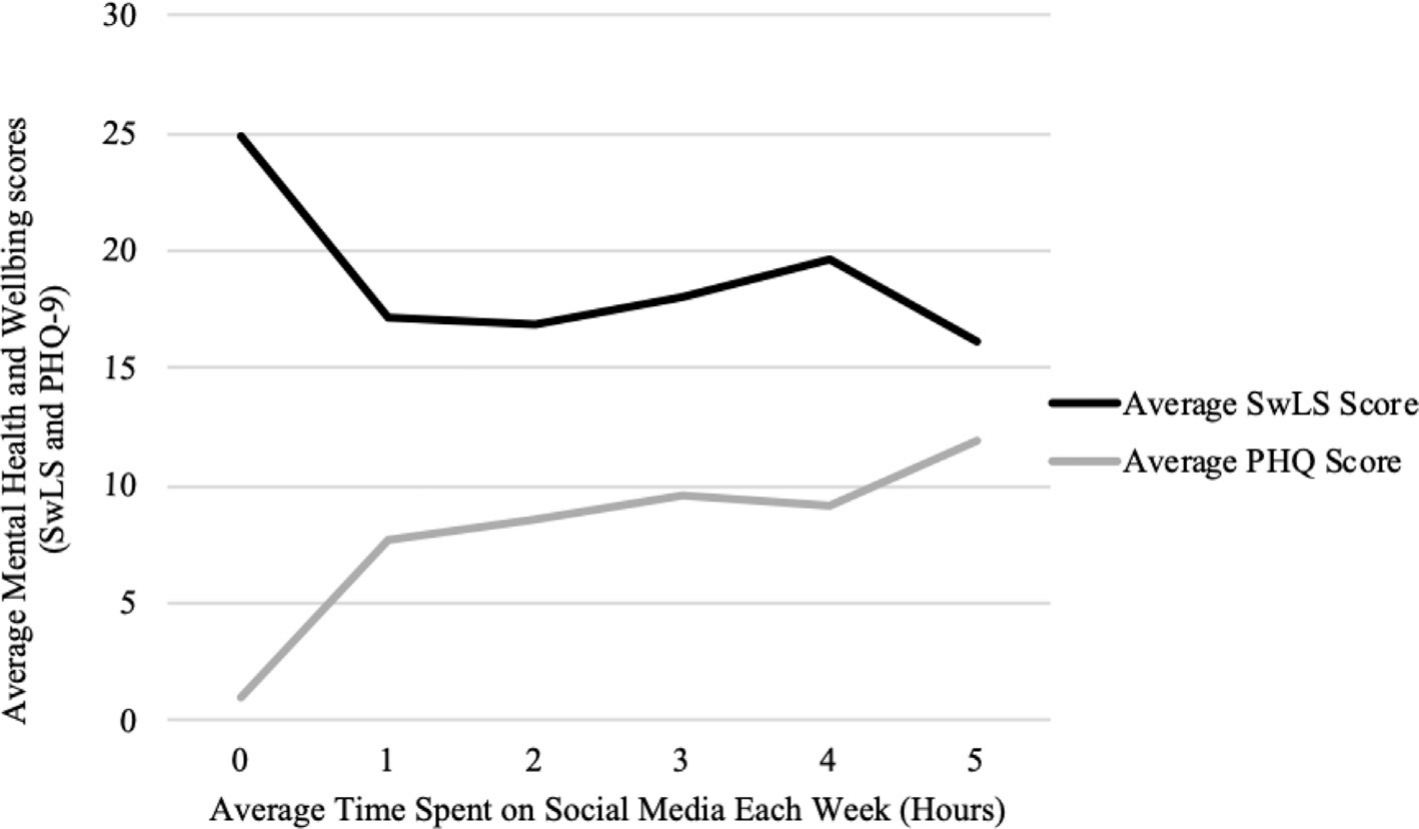
Relationship between the number of hours spent using social media per week and average scores on wellbeing and mental health measures (PHQ-9 and SwLS).

Data were also analysed to investigate whether there was a relationship between the time spent (hours) using social media and participant mental health (SwLS) and wellbeing (PHQ-9). Depicted in Figure 1, trends in the current sample demonstrated that changes to mental health and wellbeing were most apparent for SwLS and PHQ-9 scores when social media was used for approximately 5 h per week. This relationship was only significant for PHQ-9 scores ( = -.87, *n* = 6, *p* = .03).

**Figure 2:**
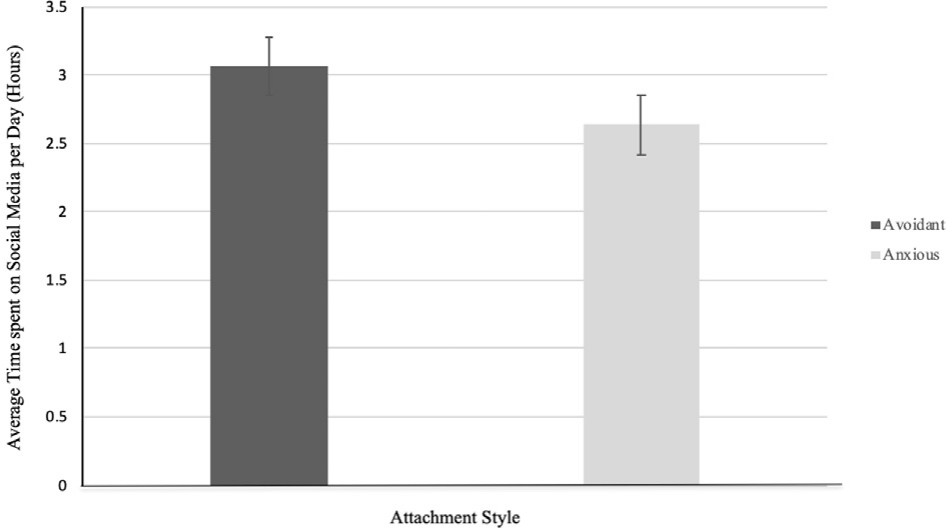
Mean (+SD) time spent on social media based on attachment style.

Data were analysed to compare the mean amount of time each attachment style spent using social media per day (see Figure 2). An independent samples t-test was conducted to compare the amount of time (rounded to the closest 15 min) that avoidantly and anxiously attached individuals spent using social media each day. Avoidantly attached participants reported spending more time using social media (M = 3.06, SD = 2.70) in comparison to anxiously attached participants (*M* = 2.63, *SD* = 1.96), though this finding was statistically non-significant; *t*(122) = .97, *p* = .33.

An independent samples t-test was also conducted to investigate the difference between participant mental health (SwLS) and wellbeing (PHQ-9) for both avoidant and anxious attachment styles. From this, a non-significant difference was observed between avoidant attachment style (*M* = 17.85, *SD* = 6.33) and anxious attachment style (*M* = 17.78, *SD* = 5.67) on the SwLS; *t*(123) = 0.07, *p* = .947. In comparison, avoidantly attached participants scored significantly higher on the PHQ-9 (*M* = 9.75, *SD* = 7.02) than anxiously attached participants (*M* = 6.98, *SD* = 4.83); *t*(123) = 2.43, *p* = .02.

Zero-order correlations were undertaken to explore relationships between variables (see Table 1 for relationships between variables and associated descriptives). PHQ-9 scores were negatively correlated with satisfaction with life (*r* = -.57, *p* < .001), and positively with levels of anxious (*r* = .54, *p* < .001) and avoidant attachment styles (*r* = .31, *p* < .001). Higher anxious and avoidant attachment styles were both related to lower satisfaction with life (*r* = -.37, *p* < .001 and *r* = -.44, *p* < .001, respectively). There was a positive relationship between levels of anxious and avoidant attachment styles (*r* = .37, *p* < .001). Problematic social media use was positively correlated with PHQ scores (*r* = .40, *p* < .001) and both dimensions of attachment style (*r* = .57, *p* < .001 and *r* = .19, *p* = .034). It was also negatively related to satisfaction with life (*r* = -.20, *p* = .032).

**Table 1:** Zero order (Pearson r) between key variables. Means and standard deviations in parentheses.

		M (SD)		(2)	(3)	(4)	(5)
(1)	Psychological wellbeing	8.71 (6.34)		-.57∗∗	.54∗∗	.31∗∗	.40∗∗
(2)	Satisfaction with Life Scale	17.73	(5.99)	–	-.37∗∗	-.44∗∗	-.20∗
(3)	Anxious Attachment	36.50	(21.55)		–	.37∗∗	.57∗∗
(4)	Avoidant Attachment	39.84	(21.75)			–	.19∗
(5)	Problematic Social Media Use	30.50	(18.60)				–

Notes: ∗∗ = p < .001, and ∗ = p < .050.

Zero-order correlations were also conducted to explore relationships between variables while controlling for both age and gender (see Tables 2 and 3). From this, we conclude that there was little effect shown for the influence of age and gender as covariates.

**Table 2:** Zero order (Pearson r) between key variables while controlling for age. Means and standard deviations in parentheses.

		M (SD)		(2)	(3)	(4)	(5)
(1)	Psychological wellbeing	8.71 (6.34)		-.55∗∗	.49∗∗	.31∗∗	.31∗∗
(2)	Satisfaction with Life Scale	17.73	(5.99)	–	-.34∗∗	-.44∗∗	-.15
(3)	Anxious Attachment	36.50	(21.55)		–	.37∗∗	.53∗∗
(4)	Avoidant Attachment	39.84	(21.75)			–	.18∗
(5)	Problematic Social Media Use	30.50	(18.60)				–

Notes: ∗∗ = p < .001, and ∗ = p < .050.

**Table 3:** Zero order (Pearson r) between key variables while controlling for gender. Means and standard deviations in parentheses.

		M (SD)		(2)	(3)	(4)	(5)
(1)	Psychological wellbeing	8.71 (6.34)		-.56∗∗	.53∗∗	.30∗∗	.40∗∗
(2)	Satisfaction with Life Scale	17.73	(5.99)	–	-.40∗∗	-.43∗∗	-.18∗
(3)	Anxious Attachment	36.50	(21.55)		–	.36∗∗	.57∗∗
(4)	Avoidant Attachment	39.84	(21.75)			–	.18∗
(5)	Problematic Social Media Use	30.50	(18.60)				–

Notes: ∗∗ = p < .001, and ∗ = p < .050.

### 3.1 Moderation analyses^1^

To test the moderating effects of both anxious and avoidant attachment styles, moderation analysis was undertaken using Model 2 in PROCESS macro (Hayes, 2018). This tested the relationship between problematic Internet use and each outcome variable, moderated by levels of both anxious attachment and anxiety attachment levels. Separate models were tested for SwLS and User wellbeing (PHQ-9) outcome measures. Five thousand bootstrap samples were taken, and confidence intervals of 95% tested.

### 3.2 Satisfaction with life

The overall model included 124 participants and was statistically significant, *R*^2^ = .26, *F*(5,118) = 8.29, *p* <.001. The relationship between problematic social media use (GPUIS) and user mental health (SwLS) was not significant (*b*^*r*^ = 0.01, *t* = 0.05, CIs = -0.39, 0.41). Avoidant attachment style significantly moderated when SwLS was the outcome variable (*b*^*r*^ = -0.52, *t* = 3.52, p <.001, CIs = -0.81,-0.23), however, the interaction effect was non-significant (*b*^*r*^ = -0.10, *t* = 1.45, CIs = -0.04,0.24).The effects of anxious attachment style as a moderator were also non-significant (b^r^ = -.03, *t* = 0.17 CIs = - 0.40,0.33) as was the associated interaction effect (b^r^ = -0.09, *t* = 1.36, CIs = -0.23,0.04). As the avoidant and anxious attachment style interaction effects were both non-significant, the conditional effects were not analysed further.

### 3.3 Psychological wellbeing

Avoidant attachment style had a significant positive relationship with PHQ-9 scores (*b*^*r*^ = 2.02, *t* = 2.65, *CI*s = 0.51,3.53). The interaction between avoidant attachment style and PHQ-9 was also statistically significant (*b*^*r*^ = -0.71, *t* = -2.03, CIs = -1.42,-0.12). Anxious attachment style did not significantly affect PHQ-9 scores (*b*^*r*^ = 0.65, *t* = 0.68, CIs = -1.24,2.54). The interaction between anxious attachment style and PHQ-9 was non-significant at the *p* <.05 level (*p* = .051;*b*^*r*^ = -0.71, *t* = 1.97, CIs = - <0.01,1.42). Due to the significant interaction effects of attachment style and the relationship between problematic social media use and PHQ-9 scores, condition effects were further broken down (see Table 4) to test the a-prori predictions. This analysis demonstrated that the relationship between problematic Internet use and mental health measured via the PHQ-9 was significantly greatest (*p* = <.001) when anxious attachment style was high and avoidant attachment style was low (*b* = 2.62, *t* = 2.97, CIs = 0.88,4.40).

**Table 4:** Effects of attachment style on general wellbeing (PHQ-9).

		Mean anxious attachment		
		Low	Medium	High
*Mean avoidant attachment*	Low	0.97 (-0.50,2.45)	1.77 (0.39,3.16)	2.62 (0.88,4.37)
	Medium	-0.13 (-1.43,1.17)	0.68 (-0.26,1.62)	1.52 (0.32,2.73)
	High	-1.06 (-2.80,0.69)	-0.25 (-1.60,1.09)	0.59 (-0.79,1.97)

Note: Positive effects represent participants achieving higher scores on the PHQ-9 scale. Effects are calculated at low (M-1SD) Medium (M) and High (M+1SD) levels of attachment style as a moderator.

## 4 Discussion

With the digitalisation of social interaction, it is important to identify how mental health is affected in a socially inclined species (Fiske, 2018; Frith and Frith, 2010). Previous research has provided inconclusive findings with some studies outlining benefits and others highlighting problematic outcomes associated with SNS use (Campbell et al., 2006). Past research has reduced problematic SNS use to only one or few predicting variables (i.e. addiction or personality issues) and reducing analysis to one or few online platforms (i.e. as Facebook or Instagram) (Hart et al., 2015; Lin, 2015; Nitzburg and Farber, 2013; Oldmeadow et al., 2013). Research has also typically reduced mental health and wellbeing to the symptomology of depression or anxiety exclusively; ignoring a magnitude of other outcomes, symptomologies and possible co-morbidities.

The aim of the current study was to investigate whether the effects of SNS use on mental health and wellbeing were moderated by attachment style. It was hypothesised that user mental health and wellbeing would be positively correlated with problematic SNS use when users reported high attachment anxiety and low avoidance. A significant negative relationship was found between problematic SNS use and levels of psychological wellbeing (measured via PHQ-9) and satisfaction with life (SwLS), providing evidence in line with past research (Nowland et al., 2018; Oldmeadow et al., 2013; Valkenburg and Peter, 2007).

### 4.1 Theoretical and practical implications

This study provides evidence to support previous research that argued for the existence of negative outcomes from problematic SNS use (Kraut et al., 1998). The current findings fit with the assumption that avoidantly-attached individuals could be more likely to use social media more abundantly to alleviate feelings of loneliness (Bessière et al., 2008), counteracting assumptions that social connectedness is not desired by avoidantly-attached individuals due to fear of disclosure and feeling undeserving (see Marshall et al., 2018).

Results can be interpreted to suggest that avoidantly-attached individuals use SNS to alleviate distress (Chen, 2019). In line with the buffer hypothesis (Caplan et al., 2009), theories of deindividualisation (Diener et al., 1980; Klein et al., 2007; Reicher et al., 1995; Silke, 2003) and the belongingness hypothesis (Baumeister and Leary, 1995) it is suggested that SNS use can be intended to alleviate unwanted distress for avoidantly-attached individuals. However, as the current study found that wellbeing scores (PHQ-9) were higher for those with an avoidant attachment style, evidence suggests outcomes can often also counteract this intention (Caplan et al., 2009).

One novel finding is the position of attachment style within the relationship between problematic SNS use and user mental health and wellbeing. Individuals who reported attachment avoidance were shown to experience significantly higher PHQ-9 scores, which suggests more symptoms of depression. Further, PHQ-9 scores were highest when avoidant attachment style was low. This is in line with traditional models of attachment style that state that avoidantly attached individuals lack the desire to form social bonds, even with online anonymity (McKenna and Bargh, 1999). Ringing true to the displacement hypothesis (Valkenburg and Peter, 2007), this finding infers that avoidantly-attached individuals view even online relationships as unworthy and could suggest that wellbeing outcomes to social networking will illicit negative responses, challenging previous explanations like the buffer hypothesis (Caplan et al., 2009). This finding also offers explanation to why avoidant attachment style was more indicative of poor wellbeing because avoidantly-attached individuals have reported to be less open online and maintain more negative preconceptions of online relationships (Oldmeadow, 2013). Therefore, it is speculated that avoidantly-attached individuals could be indifferent about social capital and capable of shutting down their attachment centers (Mikulincer et al., 2003), which results in reports of loneliness, isolation, depression and other psychological delinquencies, due to social media use (Hart et al., 2015).

The finding that wellbeing scores (PHQ-9) were most affected when participant attachment style was highly anxious and low avoidant contributes to the understanding that anxiously attached individuals are more likely to experience negative effects on their wellbeing following social media use. This finding provides a direct contrast to previous research that stipulated the benefits of using social media as the provision of a safe place to grow social capital and perceived connectedness that social media possesses (Nitzburg and Farber, 2013; Worsley et al., 2018). This finding also refutes past theories that have been used to explain the benefits that anxiously attached individuals can experience from social media use. For example, the Social Identity model of Deindividuation Effects theory (SIDE) (Klein et al., 2007).

Conversely, this finding provides support for the association between avoidant attachment style and low self-reports of wellbeing (Wei et al., 2005). Therefore, the current research presents evidence for the role of attachment style as a moderating variable between problematic social media and user wellbeing as it outlines how differences in attachment style can affect wellbeing to present different outcomes in different users.

Although we cannot infer from the current study if problematic SNS use is on the whole a cause of problematic wellbeing and mental health, the findings are useful to predict individuals who could be most at risk. The current findings can be generalised to convey that attachment style serves social skills similarly online as it does offline and that individuals with certain social orientations will be more drawn to using social media than others and for different intentions (Sherrell and Lambie, 2018). Current findings are useful to identify that avoidantly-attached individuals are likely to turn to social media to alleviate unwanted distress; however, they seem unlikely to benefit from doing so and, as highlighted in the current study, can suffer from decreasing levels of wellbeing as a result. On the other hand, the current findings suggest that anxiously attached individuals could be more successful at benefitting from using social media unless they also exhibit low avoidant attachment.

### 4.2 Limitations and future research

The present study has several limitations that may have affected analyses and can be addressed through further research. Firstly, despite the attempt to generalise materials to be inclusive of a larger population, this study fails to acknowledge covariates that may have affected the extent to which attachment style moderates. Commonly, previous research has revealed a range of correlates to include neurobiology (Troisi et al., 2017), cognitive and affective processes (Schneider-Hassloff et al., 2015). However, more recently the organisational effect of sex on attachment style has also been explored more extensively (Del Giudice and Angeleri, 2016). According to Haydon et al. (2014), woman display lower levels of avoidance and higher levels of anxiety, whereas male participants present paradoxically. This is in line with the present study's findings' where wellbeing (PHQ-9) was moderated more when anxious attachment was high and avoidant attachment was low, which could be expected due to female participants forming 80% of the sample. Haydon et al. (2014) suggested that such sex differences are particularly significant within the dimensional coding of attachment style.

Likewise, relationship status was not measured for in the current study. The current study focused on traditional definitions of attachment style (parental) rather than romantic attachment style. Some studies have commented on the similarity between parental attachment style and romantic relationships (see Akao et al., 2017; Potard et al., 2014). Previously, it has also been speculated that shifts in attachment style within these two contexts are possible for insecurely attached individuals over time (Kamenov and Jelić, 2005). Therefore, it could be argued that the current study could be diminishing the true effects of attachment style. With this in mind, future research should explore how attachment style could be affected further by covariates such as sex as an individual difference as well as varying levels of attachment style (parental or relationship). Other covariates that could also be controlled for include socio-demographic variables that have been reported to affect attachment style such as age (Chopik et al., 2019; Jones et al., 2018), gender (Haydon et al., 2014) and nationality (Agishtein and Brumbaugh, 2013). The size of the sample and lack of a-priori hypothesizing led to us not conducting additional analysis in the current study around these issues.

A similar limitation concerns the attachment styles of the achieved sample. Upon analysis, 60% of the sample were found to be avoidantly-attached. With previous scholars commenting that avoidantly-attached individuals were generally more pessimistic and more likely to make negative appraisals of life (Mikulincer and Shaver, 2019), it should be assumed that the sample does not adequately represent the general population. As such, future research should control for attachment style to ensure a representative mix. The findings of the current research should only be used as a guide for future research and not as a fixed generalisation. As such, the cross-sectional denature of the findings does not allow for a causal role to be established. As this is a novel study, procedural replications are advised before results are generalised.

## 5 Conclusion

The current study provides preliminary evidence for the role of attachment style as a moderator between social media use and user mental health and wellbeing, providing an insight into how online social capital is affected developmentally by parenting style, and how mental health is collaterally affected. The current findings suggest that individuals who are high in anxious attachment and low in attachment avoidance are more likely to report significantly higher levels of depressive symptomology from problematic SNS use.

## Declarations

### Author contribution statement

L. Young: Conceived and designed the experiments; Performed the experiments; Analyzed and interpreted the data; Contributed reagents, materials, analysis tools or data; Wrote the paper.

D. Frings: Conceived and designed the experiments; Analyzed and interpreted the data; Contributed reagents, materials, analysis tools or data; Wrote the paper.

D. C. Kolubinski: Analyzed and interpreted the data; Wrote the paper.

### Funding statement

This research did not receive any specific grant from funding agencies in the public, commercial, or not-for-profit sectors.

### Competing interest statement

The authors declare no conflict of interest.

### Additional information

No additional information is available for this paper.

## Footnotes

1 OLS regressions were conducted to investigate whether problematic social media use predicts mental health once all other variables were controlled. Results did not differ from those reported and so it was assumed that problematic social media use predicts mental health outcomes in the current study.
